# The Effect of Zeolite Features on the Dehydration Reaction of Methanol to Dimethyl Ether: Catalytic Behaviour and Kinetics

**DOI:** 10.3390/ma13235577

**Published:** 2020-12-07

**Authors:** Enrico Catizzone, Emanuele Giglio, Massimo Migliori, Paolo C. Cozzucoli, Girolamo Giordano

**Affiliations:** 1 Laboratory of Catalysis and Industrial Chemistry, University of Calabria, via P. Bucci, 87036 Rende, Italy; massimo.migliori@unical.it (M.M.); ggiordaunical@yahoo.it (G.G.); 2 Department DESF, University of Calabria, via P. Bucci, 87036 Rende, Italy; cozzucoli@unical.it

**Keywords:** dimethyl ether, zeolites, acidity, crystal size, kinetic analysis

## Abstract

The synthesis of dimethyl ether (DME) is an important step in the production of chemical intermediate because it is possible to prepare it by direct hydrogenation of CO_2_. This paper reports the effect of different zeolitic frameworks (such as: BEA, EUO, FER, MFI, MOR, MTW, TON) on methanol conversion, DME selectivity and catalyst deactivation. The effect of crystal size, Si/Al ratio and acidity of the investigated catalysts have been also studied. Finally, the kinetic parameters (such as: ∆H, ∆S and ∆G) have been evaluated together with pre-exponential factor and activation energy for catalysts with FER and MFI structure topology.

## 1. Introduction

The climate change due to the global warming is mainly caused by the emission of carbon dioxide, a greenhouse gas. A reduction in CO_2_ emission is imperative in order to avoid the dangerous effects of global warming. One possible pathway consists of using CO_2_ as a raw material in organic chemical reactions. Different ways for the utilisation of CO_2_ as a carbon source have been proposed, such as carbonilation reactions, dry reforming of methane and direct hydrogenation of CO_2_ to methanol or dimethyl ether (DME) [1,2,3,4,5,6]. The latter reaction offers an interesting perspective as it can push towards the production of olefins via methanol/DME. DME is the simplest ether, showings very interesting properties as substitute of diesel fuel (e.g., high cetane number, low ignition temperature and soot-free exhaust) [7,8,9]. DME can be used also to produce aromatics and gasoline [10,11]. Furthermore, dimethyl ether shows properties similar to those of LPG (Liquefied Petroleum Gas), enabling the use of conventional transport devices fuelled by DME. In order to replace ozone-destroying chloro-fluoro-carbides (CFC) compounds, DME is also used as propellent in spray-cans. DME is an intermediate in Methanol to Olefins (MTO) processes [12]. Methanol can be synthesised via hydrogenation of CO and/or CO_2_. Hydrogen is thus a key reactant of this pathway and it can be produced in a sustainable way via electrochemical water splitting photo-catalytic reactions [13] or through the reforming of methane produced via renewable processes as anaerobic digestion of biomass (including municipal wastes) [14,15,16]. 

Zeolite materials are considered very good catalysts in acid reactions and if a transaction metals are incorporated also in redox reactions [17,18,19]. Moreover, in methanol to DME reactions zeolites show better behaviour than other material such as γ-alumina especially concerning the selectivity and activity at low temperature [20,21,22,23,24,25]. The ability of zeolitic catalysts to address the methanol to DME or MTO reaction is well summarised in several papers [26,27] and their use in direct hydrogenation of CO_2_-to-DME is confirmed in many articles available in the open literature [28,29,30,31,32,33,34,35,36,37,38].

Nanostructured materials represent an interesting option to synthesise heterogeneous catalysts involved in fuel processing [39] and several other applications related to green chemistry. Crystal size and morphology are key features for the production of nanostructured catalysts with tailored properties [40]. Nanomaterial design for environmental applications as pollutant removal and wastewater treatment has been recently investigated [41].

In this paper, the performance in methanol-to-DME reaction of different zeolitic framework structures (such as BEA, EUO, FER, MFI, MOR, MTW, TON) was compared with γ-Al_2_O_3_ (the traditional catalyst used in industrial processes). Some zeolitic catalysts are more effective in MTO or MTH (methanol to hydrocarbons). We clearly identified the better behaviour of FER and MFI zeolitic frameworks in the methanol to DME reaction by evaluating methanol conversion, DME selectivity and carbon deposition on the catalysts and by relating their superior performance to the catalyst acidity. The MFI zeolitic framework supported on membranes shows very interesting properties in the methanol to DME reaction, especially in DME selectivity [42]. This kind of zeolite also preserves its structural properties at very high pressure for the methanol intrusion [43], this means that it can operate in industrial high-pressure processes, such as hydrocracking and catalytic dewaxing. The crystal size of catalysts and the Si/Al ratio for FER type zeolite (the zeolite that shows the better parameters concerning conversion, selectivity and coke deposition) was compared in order to understand the effect of these parameters on the catalytic behaviour. Finally, a kinetics analysis of the two better zeolitic structures (FER and MFI) that show the best overall catalytic performances is presented.

## 2. Experimental 

### 2.1. Synthesis of the Investigated Samples

Several silico–aluminate zeolites with different framework types, crystal sizes and aluminium contents were synthesised by hydrothermal crystallisation in PTFE-lined stainless-steel autoclaves. Framework details of the investigated samples are reported in Table 1.

For all the FER-type samples, for the TON-type sample and for the EUO-type sample, the autoclave was under stirred conditions in a tumbling oven with a speed of 20 rpm. For the other samples, static conditions were adopted. In Table 2 the starting gel molar compositions are reported. 

All the reactants were purchased from Sigma Aldrich (Darmstadt, Germany). Sodium hydroxide or potassium hydroxide were used as the sodium or potassium source. Sodium aluminate was used as the aluminium source, except for the EU-1 and ZSM-22 samples where aluminium nitrate and aluminium sulphate, respectively, were adopted. Precipitated silica (Silica gel 60) was used as the silica source for the MFI-type, MOR, ZSM-12, and beta samples, while colloidal silica (LUDOX AS40) was used for the FER-type and ZSM-22 samples. Details on the synthesis procedure are reported in the references indicated in the Table 2. Before synthesis, the PTFE autoclaves were cleaned with hydrofluoric acid and nitric acid for 1 day in order to remove any inorganic or organic residues. Residual acids were neutralised with ammonia solution. Afterwards, the autoclaves were washed several times with distilled water. After synthesis, the samples were recovered by filtration, washed several times with distilled water and dried at 80 °C for 1 day.

The synthesised zeolite samples contain organic molecules inside the channels (except for MOR sample) and alkali metal ions (sodium or potassium). In order to remove the organics from the channels a calcination was carried out. In particular, the sample was heated in a tubular furnace up to 550 °C with a heating rate of 5 °C/min in presence of air and kept at 550 °C for 8 h. In order to exchange the sodium and potassium ions with protons, an ion exchange with NH_4_Cl 1 M solution at 80 °C for 4 h was performed, followed by a calcination at 550 °C.

### 2.2. Characterisation of the Investigated Samples

All the investigated catalysts were characterised by X-Ray powder diffraction (XRD), nitrogen porosimetry, chemical analysis, scanning electron microscopy, and temperature-programmed desorption of pre-adsorbed ammonia (NH_3_-TPD).

XRD spectra were recorded by an APD 2000 Pro diffractometer (G.N.R s.r.l.Agrate Conturbia, Novara, Italy) operating at 40 kV and 30 mA, employing CuKα radiation in the 2θ range 5–50°, with a wavelength of 1.5406 Å. 

The main textural properties (i.e., surface area and pore volume) of the investigated samples were estimated by analysis of isotherm adsorption/desorption of nitrogen in a range of relative pressure 0–0.99 P/P° at 77 K by using an ASAP 2020 instrument (Micromeritcs, Narcross, GA, USA). The specific surface area and micropore volume were estimated by the B.E.T. and t-plot model, respectively. Prior to the analysis, the samples were evacuated at 40 µHg and 300 °C for 6 h. 

Elemental analysis was performed by atomic adsorption technique with a GBC 932 spectrometer (GBC Scientific Equipment, Hampshire, IL, USA) after zeolite dissolution in HF/HNO_3_ solution. The acids purchased from Merck were suprapure grade. 

Crystal morphology and size of the synthesised samples was analysed by Scanning Electron Microscopy (SEM) on a MIRA-LMH instrument (Tescan, Brno – Kohoutovice, Brno, Czech Republic). Before the analysis, the zeolite powder was sprinkled on carbon tape mounted on an aluminium stub and metalised by graphite.

The acidity was estimated by temperature programmed desorption of ammonia with a TPD 1100 instrument (ThermoFisher Scientific, Waltham, MA, USA) by adopting the following procedure. An amount of 100 mg of dry catalyst powder (partially dried at 180 °C in a static oven for three hours in order to remove the main part of moisture that can affect the weight of the analysed sample) is loaded in a quartz tubular reactor and contained between two layers of quartz wool. The sample is then pre-treated as follows: (i) drying at 300 °C in helium flow (20 mL/min) for 30 min, followed by cooling at 150 °C (holding time: 5 min); (ii) adsorption of ammonia at 150 °C by using a diluted ammonia stream (NH_3_/He, 10% *v*/*v*) with a flow of 20 mL/min for 120 min; (iii) elimination of the physi-adsorbed ammonia by purging under helium flow (20 mL/min) for 90 min and (iv) cooling of the sample at 100 °C. Desorption of pre-adsorbed ammonia is carried out in helium flow (20 mL/min) by heating of the sample between 100 °C and 700 °C (ramp of 10 °C/min, holding time at 700 °C: 90 min). Obtained ammonia desorption profiles are deconvoluted (PeakFit 4.12, Seasolve, San Jose, CA, USA) to calculate the area (and ammonia amount) under peaks at different temperatures. 

### 2.3. Catalytic Tests and Kinetic Analysis

The methanol conversion reaction was carried out in bench-scale experimental apparatus equipped with quartz fixed-bed reactor. Nitrogen acted as carrier and its flow rate (60 NmL/min) was controlled by a mass flow controller (Bronkhorst, Ruurlo, The Netherlands). N_2_ was bubbled though liquid methanol kept at a constant temperature in a thermostatic bath (Julabo F12-ED, Julabo, Seelbach, Germany). A methanol molar fraction of 0.06 was obtained by setting the bath temperature at 8 °C. The reaction takes place in a vertical tubular reactor (i.d. 15 mm, total length 40 mm) where 70 mg of catalytic pellet (300–500 µm) were loaded on a porous septum. The reactor is located in a vertical tubular oven with a temperature controller. Before any test, the catalytic bed was treated at 240 °C under nitrogen flow. The composition of the stream leaving the reactor was analysed using GC (Agilent 7890 A, Agilent, Santa Clara, CA, USA) equipped with a specific column (J&W 125–1032, Agilent, Santa Clara, CA, USA) and an FID detector using hydrogen as carrier and fuel. During the analysis, the GC oven is heated from 35 °C to 150 °C with a thermal ramp of 10 °C/min. All data of conversion and selectivity were calculated on the basis of at least three independent measurements. The coefficient of variation was always lower than 3%.

Experimental conversion values were used to estimate “apparent” kinetic parameters through a simplified approach assuming first order reaction. The reaction rate constant can be thus expressed as a function of methanol experimental conversion according to the following equation:

(1) $$k=-\frac{F_{MeOH}}{m_{cat}\times c_{MeOH}}\times ln\left(1-{Χ}_{MeOH}\right)$$

where k, $F_{MeOH}$, $m_{cat}$, $c_{MeOH}$ and ${Χ}_{MeOH}$ are reaction rate constant, inlet methanol mole flow, catalyst load, inlet methanol concentration and conversion, respectively. Reaction rate constant can be expressed through an Arrhenius-type equation as follows:

(2) $$k=k_{0}\times exp\left(-\frac{E_{a}}{R\times T}\right)$$

where $k_{0}$ and $E_{a}$ represent pre-exponential factor and activation energy, respectively. The previous equation can be linearised through the Arrhenius plot (ln[k] vs. 1/T): from angular coefficient and intercept of the interpolating linear curve the activation energy and pre-exponential factor can be calculated.

According to the transition state theory, reaction rate constant can be also expressed as [47]:

(3) $$k=\frac{k_{B}\times T}{h}\times exp\left(\frac{\Delta {\bar{S}}^{\#}}{R}\right)exp\left(-\frac{\Delta {\bar{H}}^{\#}}{R\times T}\right)$$

where $k_{B}$ and h are the Boltzmann and Plank constants, respectively, while $\Delta {\bar{S}}^{\#}$ and $\Delta {\bar{H}}^{\#}$ are the activation entropy and enthalpy, respectively. The superscript “#” indicates that the parameters refer to the transition state. Activation enthalpy and entropy were estimated using a nonlinear least-squares regression tool.

Once the kinetic parameters are assessed, the estimation of effectiveness factor (η) for configurational diffusion can be carried out to elucidate the role of intra-particle mass diffusion limitation [48]:

(4) $$\eta =\frac{1}{\phi }\times \left[\frac{1}{th\left(3\phi \right)}-\frac{1}{3\phi }\right]$$

where ϕ is Thiele modulus:

(5) $$\phi =\frac{L}{6}\times \sqrt{\frac{k\times \rho }{D_{eff}}}$$

*L* is a characteristic length (crystallite size has been considered), *k* is the intrinsic reaction rate constant, ρ is sample apparent density and *D_eff_* is the effective diffusivity. Diffusivity was assumed equal to 8.1·10^−9^ cm^2^ s^−1^ for FER [49] and equal to 2.1·10^−8^ cm^2^ s^−1^ for MFI [50].

## 3. Results and Discussion

### 3.1. Physic-Chemical Properties of the Investigated Samples

**Figure 1:**
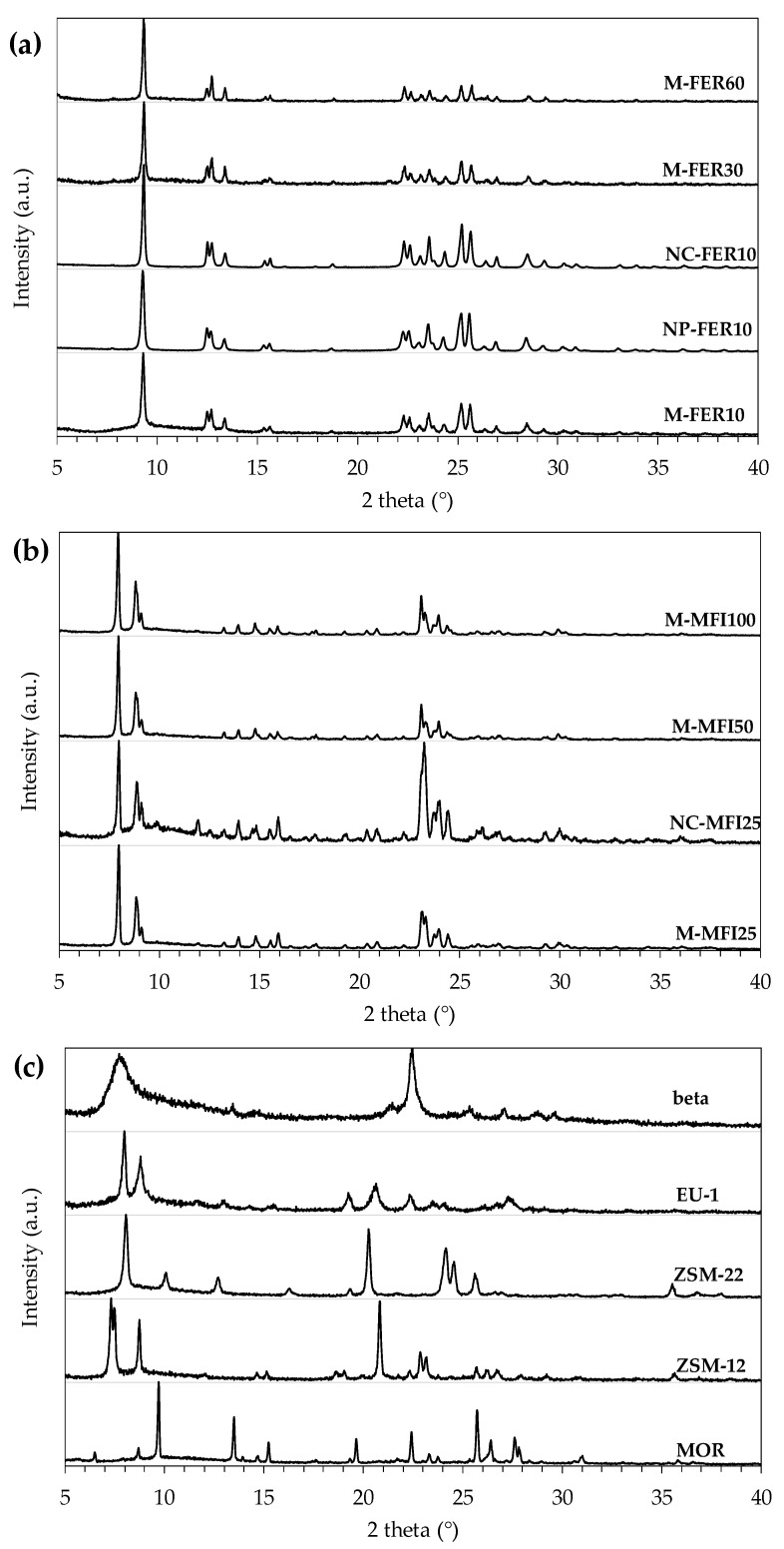
XRD of FER-type samples (**a**), MFI-type samples (**b**) and beta, EU-1, ZSM-22, ZSM-12 and MOR (**c**), after calcination.

XRD diffraction spectra of the investigated zeolites (calcined form) are reported in Figure 1. 

The obtained XRD spectra agree with the patterns reported by International Zeolite Association Structure Commission Database, and no amorphous or competitive phases are detected. Furthermore, no significant effect of crystal size or aluminium content on phase purity can be observed. In Table 3 the main physic-chemical properties of the investigated catalysts are reported. 

**Figure 2:**
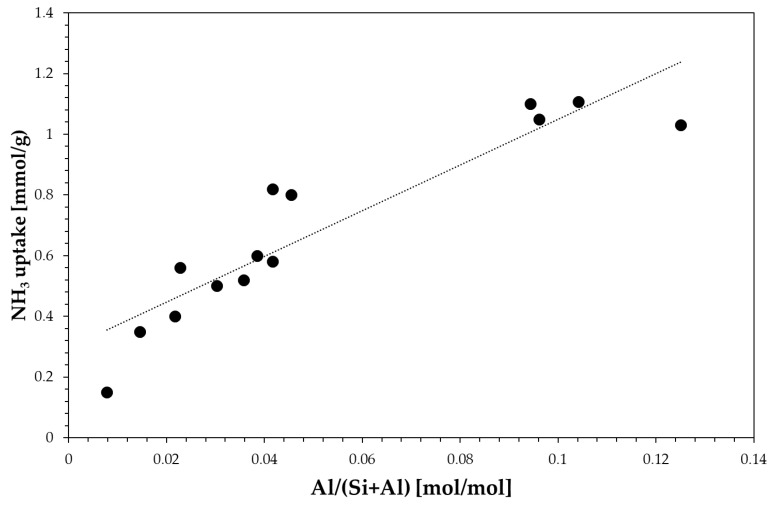
Effect of aluminium content on total acidity.

Textural properties data clearly show the effect of zeolite framework on micropore volume. For instance, zeolites with either large cavities or large channel size, such as EU-1, beta and MOR exhibit a higher micropore volume. The total acidity measured by the NH_3_-TPD technique correlates fairly well with the aluminium content as reported in Figure 2. 

The acid sites’ strength seems to be related to the zeolite framework type. For instance, all the FER-type zeolite samples possess a strong acid sites fraction, regardless if both aluminium content and crystal size. On the contrary, the acidity of all the MFI-type samples is equally distributed between weak and strong acid sites. This is an important aspect, as it suggests that strength of acid sites depends on the zeolite framework more than on the aluminium concentration, as result of a different Si-O-Al framework bridge angle. 

The crystal size measurement via SEM technique indicates that the M-FER10 sample consists of plat-like crystals with size in the range 5–10 µm. Larger crystals were obtained for M-FER30 and M-FER60. On the contrary, the addition of sodium lauryl sulphate, coupled with a decreasing crystallisation temperature, allows the attainment of FER-type samples in nanometric range. A Nanocrystal MFI-type sample (NC-MFI25) was obtained by aging of the starting synthesis mixture, while no significant effect of aluminium content on crystal size was observed, as both M-MFI25, M-MFI50 and M-MFI100 have a crystal size of about 5 µm. More details of crystal morphology as well as the SEM images of the investigated samples are discussed in the references reported in Table 2. 

### 3.2. Catalytic Tests

**Figure 3:**
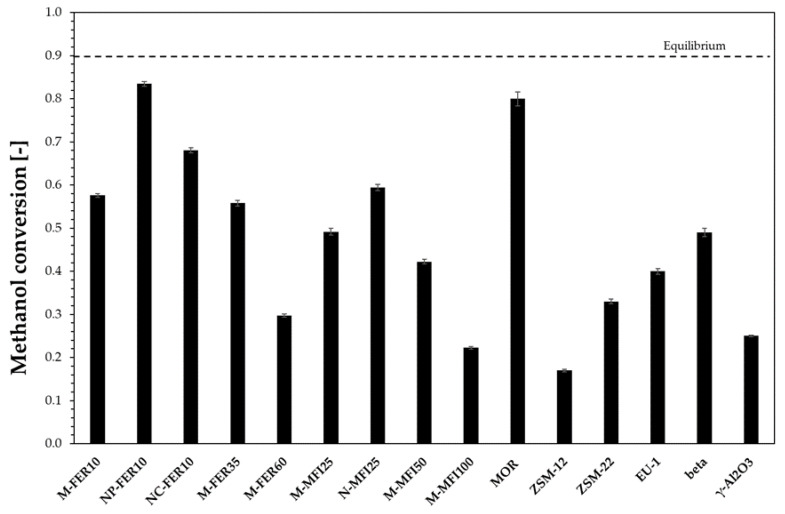
Methanol conversion at 200 °C. Dimethyl ether DME selectivity higher than 0.98 for all the samples.

The catalytic activity of the investigated samples was compared at 200 °C and results in terms of methanol conversion are reported in Figure 3. For comparison, commercial γ-Al_2_O_3_ was also tested.

All the investigated zeolites exhibit a methanol conversion higher than commercial γ-Al_2_O_3_, except for M-MFI100 and ZSM-12, in addition NP-FER10 and MOR samples are the most active samples. The observed catalytic activity may depend on several zeolite features, such as channel system, crystal size and acidity. In particular, both channel system and crystal size have an influence on diffusion of species inside the crystal, with an effect on catalyst effectiveness and then on the methanol conversion. The effect of crystal size may be discussed only for samples with the same channel system. In the case of FER-type and MFI-type samples, the beneficial effect of crystal size reduction on methanol conversion may be observed. In particular, for MFI-type samples, methanol conversion at 200 °C increases from 0.49 to 0.59 by decreasing the crystal size from 5 to 0.5 µm. A similar effect is also observed for the FER-type zeolite. Ferrierite nanocrystals exhibit a significant higher methanol conversion than micro-sized. Moreover, acidity affects methanol conversion as, in the case of both FER-type and MFI-type zeolites, methanol conversion increases as the acidity increases. In particular, in the case of MFI-type samples, methanol conversion increases from 0.22 to 0.59 by increasing the acidity from 0.15 mmol/g (M-MFI100) to 0.52 mmol/g (M-MFI25). A similar trend was also observed for FER-type zeolites. 

**Figure 4:**
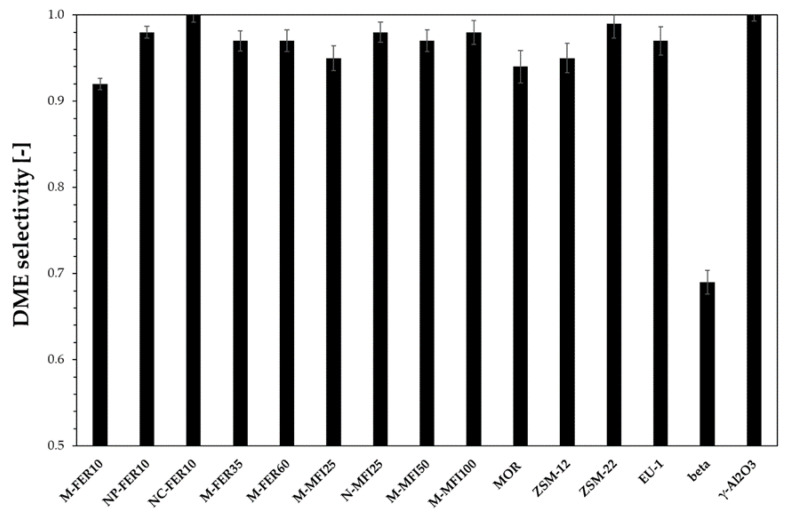
DME selectivity at 240 °C.

Figure 4 reports DME selectivity calculated at 240 °C. NC-FER10 is the most selective catalyst, with no formation of any by-product. In the other cases, light hydrocarbons were detected. For the beta sample a very low DME selectivity was calculated and more by-products were observed. In particular, the large pore size allowed oligomerisation reaction leading to the formation of C_4_-C_5_ fraction, condensing on the cold part of the reaction outstream line, determining the DME selectivity drop. Concerning MFI-type and FER-type samples, DME selectivity increases by decreasing both crystal size and acidity, suggesting that both a short reaction pathway length and a moderate acidity should be adopted to avoid consecutive reaction which leads to the formation of by-products.

**Figure 5:**
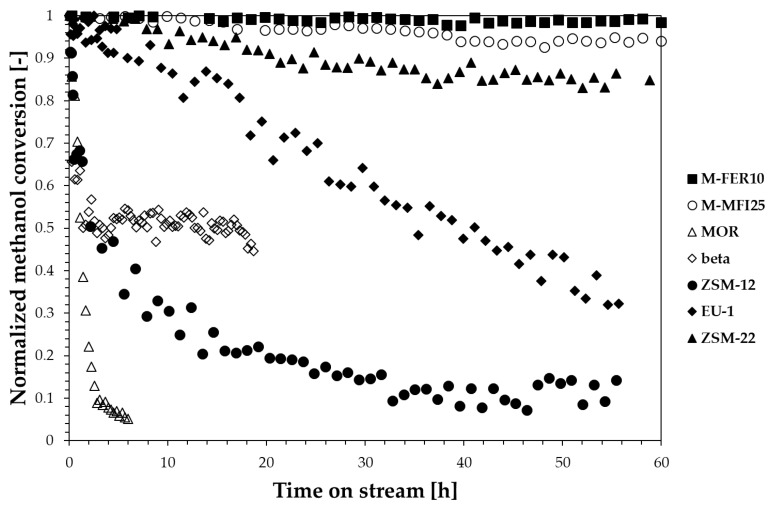
Normalised methanol conversion at 240 °C during time-on-stream tests.

Stability tests were also performed and reported in Figure 5, where the methanol conversion normalised with respect to the initial conversion value is plotted over time for all the samples (reaction temperature/time: 240 °C/60 h). 

Both FER-type and MFI-type zeolites exhibit a reliable stability during time-on-stream tests, whilst a deactivation is observed for the other zeolite structures, with a deactivation rate depending on zeolite structure. In particular, the MOR sample completely deactivates in 6 h, while a slower deactivation trend is observed for ZSM-12 and EU-1 samples. On the contrary, ZSM-22 seems to be the most stable sample among the investigated 1-dimensional structures. The beta sample shows a partial deactivation: the conversion is halved during the first minutes and then maintained. In this later case we observe the formation of hydrocarbons with more than five carbon atoms.

**Figure 6:**
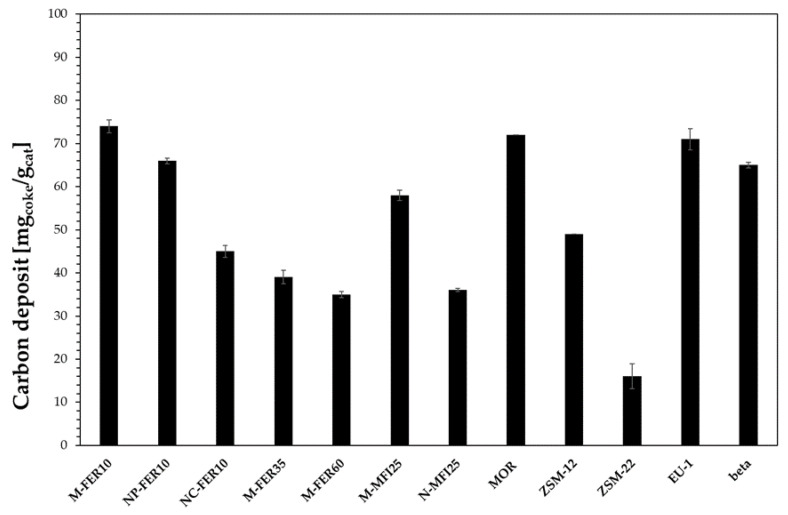
Carbon deposit after 60 h time-on-stream at 240 °C.

The deactivation of zeolites during methanol conversion reactions is usually related to the formation of heavy carbon species depositing as coke. Therefore, the zeolite framework plays a crucial role in terms of resistance to deactivation as the coke-forming species type and formation rate strongly depends on the catalyst topology. For instance, despite the similar channel openings of EU-1 and ZSM-22, EU-1 is less resistant to deactivation than ZSM-22, because of the presence of large side-pockets inside the channels which allow the formation of bulky molecules that cannot diffuse outside the crystals causing deactivation. However, when considering coke formation, other aspects, such as acidity and crystal size, should also be taken into account as reported in Figure 6 where the effect of zeolite framework, acidity and crystal size are shown. 

The beneficial effect of the reduction in crystal size on coke deposition may be observed for both FER-and MFI-type samples. In particular, the carbon deposit decreases from 74 mg/g to about 45 mg/g, and from about 60 mg/g to about 35 mg/g, by decreasing the crystal size of FER- and MFI-type zeolite, respectively, from micrometric to nanometric scale. Furthermore, for FER-type samples is also possible to observe that the carbon deposit is lower for samples with lower acidity. However, the effect of the carbon deposit on deactivation strongly depends on the zeolite framework. In fact, although a similar carbon deposit was measured for M-FER10, MOR and EU-1 samples, the deactivation is significantly different, as previously discussed. Similarly, ZSM-22 shows the lower carbon deposit, but it is less resistant to deactivation than M-FER10 and M-MFI25 samples, due to its 1-D channel system that could easily suffer a pore-blocking phenomenon.

Globally, both FER- and MFI-type zeolites seem to exhibit the best catalytic behaviour in terms of resistance to deactivation, among the investigated zeolite structure, while their activity and selectivity may be tuned by controlling either crystal size or acidity. For these reasons, the kinetic analysis was focused on these two structures, with the aim to assess the effect of crystal size and acidity on kinetic parameters.

### 3.3. Kinetic Analysis on MFI- and FER-Type Zeolites

Table 4 summarises the estimated kinetic parameters for MFI- and FER-type zeolites. As expected, the three presented parameters are in the same order of magnitude for all the investigated catalysts. Activation entropy always has a negative value. As a general trend, FER-type samples present lower values for activation energy, enthalpy, and entropy, except for the M-MFI100 sample (with kinetic parameters closer to those for FER-type). FER-type zeolites thus show both enthalpy and entropy barriers slightly lower than the MFI-type, suggesting that dimethyl ether formation is more favoured on the former ones.

Table 5 reports the calculated effectiveness factor for the investigated samples at different reaction temperatures. Besides the absolute effectiveness values, normalised (with respect to 140 °C) ones have also been reported to underline the relative effectiveness drop due to a temperature increase.

Since temperature increase favourably impacts on reaction rates, the Thiele modulus is consequently reduced (Equation (5)) resulting in a lower effectiveness factor (Equation (4)) that also depends on the crystal. In the case of nano-sized zeolites (both FER- and MFI-type), the effect of temperature on the effectiveness drop is weaker and almost no mass transfer limitation occurs, even at 200 °C, and the calculated value of effectiveness remains close to one for all the investigated temperature ranges. Micro-sized samples present a steeper resistance to mass transfer when the temperature increases, as well as considerably lower values for effectiveness. This is a clear and direct consequence of the characteristic length (L) impact on Thiele modulus and thus on the effectiveness calculation. Reported results show the importance of the crystal size reduction to reduce mass transfer limitations, especially when increasing reaction temperature.

## 4. Conclusions

The reported results clearly show the better behaviour of FER and MFI type catalysts in the methanol to DME reaction especially in terms of selectivity, stability of the catalyst and carbon species formation. The selectivity is neither strongly affect by the zeolitic framework nor by the crystal size and Si/Al ratio, with the exclusion of the beta type zeolite that favours the formation of higher hydrocarbons. On the contrary, conversion, stability and coke formation are strongly affected by zeolitic framework and acidity of the catalysts. For example, MOR, that possesses good properties in conversion and selectivity, shows a rapid deactivation and a higher coke formation.

The ZSM-22-type catalyst deserves further investigation, as it shows high selectivity, good stability, and very low carbon deposit formation. A reduction in crystal size together with an increasing specific surface area can be a starting point for further studies on this sample.

Finally, kinetic investigation shows a higher activation energy for MFI catalysts compared to the FER-type. Effectiveness factor results clearly indicate the strong effect of the catalysts’ crystal size. As a matter of fact, both nano-FER and nano-MFI samples show an effectiveness factor close to one in the investigated temperature range, whilst catalysts with larger crystals present lower values due to higher resistance to the mass transfer.

## Figures and Tables

**Table 1:** Structure details of the investigated zeolite samples.

Sample	Topology	Channel Orientation	Membered Rings	Channel Openings (Å)
ZSM-22	TON	1D	10	4.6 × 5.7
EU-1	EUO	1D	10	4.1 × 5.4
ZSM-22	MTW	1D	12	5.6 × 6.0
MOR	MOR	1D	12//8	6.5 × 7.0 < > 2.6 × 5.7
M-FER10 NP-FER10 NC-FER10 M-FER30 M-FER60	FER	2D	10 × 8	4.2 × 5.4 < > 3.5 × 4.8
M-MFI25 NC-MFI25 M-MFI50 M-MFI100	MFI	3D	10	5.1 × 5.5 < >5.3 × 5.6
beta	BEA	3D	12	6.6 × 7.7 < > 5.6 × 5.6

**Table 2:** Gel composition, temperature and time of crystallisation of the samples.

Sample Name	Framework	Synthesis Molar Gel Composition	Crystallisation		Ref.
			Temperature (°C)	Time (h)	
M-FER10	FER	0.6 C_4_H_9_N * − 0.08 Na_2_O − 0.05 Al_2_O_3_ − 1 SiO_2_ − 20 H_2_O	180	120	[44]
NP-FER10	FER	0.6 C_4_H_9_N * − 0.015 NaC_12_H_25_SO_4_ * − 0.08 Na_2_O − 0.05 Al_2_O_3_ − 1 SiO_2_ − 20 H_2_O	180	60	[44]
NC-FER10	FER	0.6 C_4_H_9_N * − 0.015 NaC_12_H_25_SO_4_ * − 0.08 Na_2_O − 0.05 Al_2_O_3_ − 1 SiO_2_ − 20 H_2_O + 3% wt of seeds **	160	60	[44]
M-FER30	FER	2 C_5_H_5_N * − 0.0575 Na_2_O − 0.017 Al_2_O_3_ − 1 SiO_2_ − 25 H_2_O	160	120	[25]
M-FER60	FER	2 C_5_H_5_N * − 0.0575 Na_2_O − 0.008 Al_2_O_3_ − 1 SiO_2_ − 25 H_2_O	160	120	[25]
M-MFI25	MFI	0.10 Na_2_O − 0.08 C_12_H_28_NBr * − 0.02 Al_2_O_3_ − 1 SiO_2_ − 20 H_2_O	170	120	[35]
M-MFI50	MFI	0.10 Na_2_O − 0.08 C_12_H_28_NBr * 0.01 Al_2_O_3_ − 1 SiO_2_− 20 H_2_O	170	120	[35]
M-MFI100	MFI	0.10 Na_2_O − 0.08 C_12_H_28_NBr * − 0.005 Al_2_O_3_ − 1 SiO_2_ − 20 H_2_O	170	120	[35]
NC-MFI25	MFI	0.10 Na_2_O − 0.08 C_12_H_28_NBr * 0.02 Al_2_O_3_ − 1 SiO_2_ − 20 H_2_O	170	90	[45]
MOR	MOR	0.20Na_2_O − 0.02Al_2_O_3_ − 1.0SiO_2_ − 20H_2_O	170	120	[46]
ZSM-12	MTW	0.1 N_2_O − 0.2 C_7_H_18_NBr * − 0.01 Al_2_O_3_ − 1 SiO_2_ − 20 H_2_O	140	150	[26]
ZSM-22	TON	0.140 K_2_O − 0.3 C_8_H_20_N_2_ * − 0.011 Al_2_O_3_ − 1 SiO_2_ − 40 H_2_O	160	80	[26]
EU-1	EUO	0.3 Na_2_O − 0.15 C_12_H_30_N_2_Br_2_ * − 0.017 Al_2_O_3_ − 1 SiO_2_ − 45 H_2_O	160	340	[26]
beta	BEA	0.10 Na_2_O − 0.2 C_8_H_21_NO * − 0.02 Al_2_O_3_ − 1 SiO_2_ − 10 H_2_O	150	120	[46]

* Template chemical name. C_4_H_9_N: pyrrolidine; NaC_12_H_25_SO_4_: sodium lauryl sulphate; C_5_H_5_N: pyridine; C_12_H_28_NBr tetrapropyl ammonium bromide; C_8_H_21_NO: Tetraethylammonium hydroxide; C_12_H_30_N_2_Br_2_: hexamethonium bromide; C_8_H_20_N_2_: 1,8-Diaminooctane; C_7_H_18_NBr: Triethylmethylammonium bromide. ** seeds of H-form NP-FER10 sample

**Table 3:** Main physic–chemical properties of the catalysts.

Sample	Specific Surface Area ^a^ (m^2^/g)	Micropore Volume ^b^ (cm^3^/g)	Mesopore Volume ^b^ (cm^3^/g)	Si/Al ^c^ (mol/mol)	Total Acidity ^d^ (mmol/g)	Strong Acid Sites Fraction ^e^ (-)	Crystal Size (µm)
M-FER10	332	0.136	0.086	9.6	1.10	0.70	5–10
NP-FER10	314	0.125	0.093	8.6	1.12	0.72	0.1–0.5
NC-FER10	304	0.122	0.071	9.4	1.10	0.70	<0.1
M-FER30	272	0.108	0.065	23	0.82	0.77	10–20
M-FER60	275	0.110	0.054	45	0.40	0.78	10–20
M-MFI25	386	0.126	0.073	27	0.52	0.58	~5
NC-MFI25	371	0.124	0.074	23	0.58	0.52	0.1–0.5
M-MFI50	316	0.124	0.070	68	0.35	0.55	~5
M-MFI100	382	0.101	0.112	127	0.15	0.54	~5
MOR	348	0.152	0.028	7	1.03	0.74	5–10
ZSM-12	294	0.115	0.031	32	0.50	0.82	2–3
ZSM-22	210	0.074	0.104	43	0.56	0.68	5–10
EU-1	384	0.146	0.061	21	0.80	0.72	<1
beta	468	0.202	0.148	25	0.60	0.58	<1

^a^: estimated by B.E.T model. ^b^: micropore volume estimated by t-plot model, mesopore volume calculated as V_tot_-V_micropore_ (V_tot_: the total volume adsorbed at P/P°=0.99). ^c^: measured by atomic absorption spectroscopy. ^d^: measured from temperature-programmed desorption of pre-adsorbed ammonia (NH3-TPD) measurements. ^e^: estimated from NH3 desorbed above 300 °C.

**Table 4:** Kinetic parameters of investigated samples.

Sample	$E_{a}\;(\mathrm{kJ}\cdot {\mathrm{mol}}^{-1})$	$\Delta {\bar{H}}^{\#}\;(\mathrm{kJ}\cdot {\mathrm{mol}}^{-1})$	$\Delta {\bar{S}}^{\#}\;(J\cdot {\mathrm{mol}}^{-1}\cdot K^{-1})$
M-FER10	60.4	49.7	−175.0
NP-FER10	58.2	51.2	−165.8
NC-FER10	61.7	47.6	−177.1
M-FER30	52.4	45.0	−185.6
M-FER60	52.3	47.2	−187.9
M-MFI25	105.5	70.7	−132.6
NC-MFI25	73.0	60.1	−152.7
M-MFI50	82.8	72.7	−136.7
M-MFI100	70.7	57.7	−174.7

**Table 5:** Effectiveness factor at different reaction temperatures. The normalised effectiveness factor with regard to the value obtained at 140 °C is reported in brackets.

Sample	Effectiveness Factor			
	140 °C	160 °C	180 °C	200 °C
M-FER10	0.2102	0.1017	0.0504	0.0260
	(1.000)	(0.4840)	(0.2396)	(0.1237)
NP-FER10	0.9916	0.9817	0.9629	0.9298
	(1.000)	(0.9900)	(0.9711)	(0.9377)
NC-FER10	0.9994	0.9987	0.9972	0.9944
	(1.000)	(0.9993)	(0.9978)	(0.9950)
M-FER30	0.0483	0.0243	0.0129	0.0072
	(1.000)	(0.5025)	(0.2664)	(0.1485)
M-FER60	0.1099	0.0566	0.0304	0.0170
	(1.000)	(0.5149)	(0.2763)	(0.1551)
M-MFI25	0.9297	0.7412	0.3924	0.1508
	(1.000)	(0.7973)	(0.4221)	(0.1622)
NC-MFI25	0.9990	0.9973	0.9935	0.9853
	(1.000)	(0.9983)	(0.9945)	(0.9863)
M-MFI50	0.9490	0.8524	0.6500	0.3883
	(1.000)	(0.8983)	(0.6850)	(0.4092)
M-MFI100	0.9609	0.9018	0.7826	0.5928
	(1.000)	(0.9385)	(0.8144)	(0.6170)
